# Prevalence, clinical features, and laboratory predictors of autoimmune hepatitis in systemic sclerosis: A retrospective single-center cohort study

**DOI:** 10.1007/s10067-026-08014-0

**Published:** 2026-03-06

**Authors:** Zeynel Abidin Akar, Dilan Yildirim

**Affiliations:** 1 Division of Rheumatology, Department of Physical Therapy and Rehabilitation, Faculty of Medicine, Dicle University, Kıtılbıl Neighborhood, Sur, Diyarbakır, Türkiye; 2 Department of Physical Therapy and Rehabilitation, Faculty of Medicine, Dicle University, Diyarbakır, Türkiye

**Keywords:** ALT, Autoimmune hepatitis, Firth regression, Overlap syndrome, ROC analysis, Systemic sclerosis

## Abstract

**Background:**

Liver involvement, particularly autoimmune hepatitis (AIH) overlap, is a rare but clinically important manifestation of systemic sclerosis (SSc). This study aimed to assess the prevalence, clinical characteristics, and diagnostic utility of laboratory parameters for identifying AIH in a cohort of patients with SSc.

**Methods:**

We retrospectively analyzed 111 patients with SSc. Clinical characteristics, autoantibody profiles, and laboratory parameters were compared between patients with and without AIH. AIH diagnosis was confirmed by liver biopsy in all cases. Given the small number of events, Firth’s penalized likelihood logistic regression was applied to identify independent risk factors. The diagnostic performance of alanine aminotransferase (ALT), aspartate aminotransferase (AST), and serum IgG levels was evaluated using receiver operating characteristic (ROC) curve analysis. Prior methotrexate (MTX) exposure was recorded, and the potential confounding effect of MTX-induced liver enzyme elevations was assessed.

**Results:**

Autoimmune hepatitis (AIH) was identified in 8 of 111 patients (7.2%). All AIH-positive patients showed interface hepatitis on liver biopsy. There were no significant differences between AIH-positive and AIH-negative patients regarding age, systemic sclerosis subtype, or presence of interstitial lung disease (all *p* > 0.05). Firth’s penalized logistic regression indicated that diffuse cutaneous SSc, anti–Scl-70 positivity, and interstitial lung disease were not independent predictors of AIH overlap. In receiver operating characteristic analysis, alanine aminotransferase (ALT) demonstrated the highest diagnostic performance (AUC 0.88, 95% CI 0.76–0.99; *p* < 0.001), with a sensitivity of 87.5% and specificity of 85.4% at a cut-off > 34.5 U/L. Aspartate aminotransferase (AST) (AUC 0.86) and serum IgG (AUC 0.74) also showed significant but lower discriminatory ability. ALT retained high specificity for AIH even among patients with prior MTX exposure, supporting its utility as a non-invasive screening tool in SSc.

**Conclusion:**

Autoimmune hepatitis is a rare overlap syndrome in systemic sclerosis that occurs independently of clinical phenotype. Routine monitoring of liver transaminases, particularly ALT, provides a reliable non-invasive screening tool. Clinicians should consider ALT elevations > 34.5 U/L as a trigger for further AIH evaluation, even in patients with prior MTX exposure.

**Table:** 

**Key Points** • *Autoimmune hepatitis (AIH) represents a clinically significant overlap in systemic sclerosis (SSc), with a prevalence of 7.2% in this cohort, indicating that it may be more common than previously reported when systematic screening is implemented. Biochemical screening rather than biopsy-only evaluation improves detection.* • *The development of AIH in SSc patients appears to be independent of disease subtype (limited vs. diffuse), specific autoantibody profiles (Anti-Scl70, ACA), or major organ involvement, such as interstitial lung disease.* • *An ALT threshold of > 34.5 U/L provides a practical “red flag” for clinicians, offering high sensitivity and specificity to prompt further diagnostic evaluation, including liver-specific autoantibody testing and liver biopsy when indicated. This threshold should be interpreted cautiously in patients with prior methotrexate (MTX) exposure, as mild ALT elevations may also reflect drug-related hepatotoxicity.* • *An ALT threshold of > 34.5 U/L provides a practical “red flag” for clinicians, offering high sensitivity and specificity to prompt further diagnostic evaluation, including liver-specific autoantibody testing and liver biopsy when indicated.* • *Because AIH can develop across the full spectrum of SSc regardless of systemic disease severity, routine monitoring of transaminases is essential for early detection and timely initiation of immunosuppressive therapy.*

## Introduction

Systemic sclerosis (SSc) is a complex and chronic autoimmune connective tissue disease characterized by multisystemic involvement, resulting from a unique triad of vascular damage, immunological activation, and widespread tissue fibrosis [1]. While the hallmark of SSc is cutaneous thickening, the prognosis is predominantly determined by internal organ involvements, most commonly the lungs, heart, and kidneys [2]. Historically, the liver was considered a rare target in SSc; however, emerging clinical data suggest that hepatic manifestations may occur more frequently than previously estimated, ranging from asymptomatic biochemical abnormalities to clinically significant conditions. Among these, the overlap of SSc with autoimmune hepatitis (AIH) represents a distinct and rare clinical entity [3]. Although the pathophysiological link between these two conditions remains partially understood, the presence of AIH in SSc patients necessitates careful clinical attention due to its potential impact on disease morbidity and the requirement for specific immunosuppressive management strategies. Routine biochemical screening of transaminases rather than biopsy-only evaluation may improve detection rates and provide earlier clinical intervention.

Autoimmune hepatitis (AIH) is characterized by elevated transaminases, hypergammaglobulinemia, and specific histological hallmarks—most notably interface hepatitis and plasma cell infiltration [4]. When AIH manifests within the clinical spectrum of systemic sclerosis (SSc), it poses significant diagnostic and therapeutic challenges [5]. Current literature reports the prevalence of this overlap syndrome to be as low as 1% to 4%, making it a rare phenomenon compared to other hepatic associations such as primary biliary cholangitis [6]. However, the relative scarcity of SSc–AIH cases often leads to its underrecognition, which is particularly concerning given the risk of progression to liver cirrhosis and end-stage liver disease if left untreated. The clinical presentation of AIH in SSc patients is frequently asymptomatic in the early stages, where biochemical markers may be the only indicators of ongoing hepatic inflammation. Furthermore, distinguishing AIH from drug-induced liver injury (DILI)—a common occurrence due to the extensive polypharmacy in SSc, including methotrexate (MTX) exposure—requires a high index of clinical suspicion and invasive confirmation via liver biopsy. Clinicians should interpret mild ALT elevations cautiously in patients with prior hepatotoxic drug exposure. Therefore, identifying reliable markers for early detection and characterizing the clinical phenotype of these patients is paramount to optimizing long-term outcomes and preventing irreversible hepatic damage.

The relationship between specific systemic sclerosis (SSc) phenotypes and the development of autoimmune hepatitis (AIH) remains a subject of ongoing debate [7]. While certain SSc-related autoantibodies and organ involvements, such as interstitial lung disease (ILD) or diffuse cutaneous involvement (dcSSc), are known to correlate with more aggressive disease courses, their predictive value for AIH overlap has not been consistently established [8]. Most existing data on SSc–AIH overlap are derived from case reports or small-scale retrospective cohorts, which limits the ability to draw definitive conclusions regarding risk profiles. Consequently, it remains unclear whether AIH occurs as a stochastic autoimmune event or is linked to a specific systemic phenotype [9]. Consideration of genetic susceptibility and HLA associations may provide additional mechanistic insight. This knowledge gap highlights the need for a comprehensive evaluation of clinical and laboratory markers to establish reliable screening strategies in this high-risk population.

Despite the significant clinical impact of autoimmune hepatitis (AIH) on systemic sclerosis (SSc) outcomes, there remains a lack of comprehensive studies addressing both clinical predictors and diagnostic thresholds of laboratory markers in this specific overlap [10]. Most of the existing literature is limited by small sample sizes or lacks a detailed evaluation of non-invasive screening tools [11]. This study was designed to address these gaps by providing a multi-faceted analysis of a well-defined SSc cohort. Our primary objective was to determine the prevalence of AIH within this population and to characterize its clinical and serological features. Furthermore, we aimed to investigate potential risk factors associated with AIH development and to rigorously evaluate the diagnostic performance of routine biochemical markers. By establishing optimal cut-off values for screening, this study seeks to provide clinicians with a practical framework for the early detection and management of AIH in patients with systemic sclerosis, ultimately contributing to improved prognostic stratification.

## Materials and methods

### Study population and ethics

This retrospective cohort study was conducted at a single tertiary referral center. We reviewed the medical records of consecutive adult patients (≥ 18 years) diagnosed with systemic sclerosis (SSc) who were followed at the Rheumatology Clinic between January 2015 and January 2025. A total of 111 patients fulfilling the 2013 American College of Rheumatology (ACR)/European League Against Rheumatism (EULAR) classification criteria for SSc were included [12]. Patients were required to have at least 12 months of clinical follow-up and available laboratory data, including routine liver function tests, to allow consistent evaluation of hepatic involvement.

Exclusion criteria were applied to ensure that hepatic involvement was specifically attributable to autoimmune hepatitis (AIH). Patients were excluded if they had:

1. Active viral hepatitis (HBV, HCV, or HIV),
2. Significant alcohol consumption,
3. Overlap with primary biliary cholangitis (PBC) or primary sclerosing cholangitis (PSC),
4. Non-alcoholic fatty liver disease with evidence of steatohepatitis, or.
5. Drug-induced liver injury (DILI), including methotrexate (MTX)-associated hepatotoxicity, when a clear temporal relationship between liver enzyme elevation and hepatotoxic medication use was established.

All SSc patients underwent routine monitoring of liver transaminases during follow-up, regardless of clinical phenotype. Patients with persistent elevation of transaminases and/or clinical suspicion of AIH were referred for hepatological evaluation. The diagnosis of AIH was confirmed in all cases by liver biopsy demonstrating interface hepatitis, in conjunction with compatible serological findings and elevated serum IgG levels, according to established international diagnostic criteria. This approach ensures that the reported prevalence reflects biopsy-confirmed AIH rather than transient or drug-related enzyme elevations.

The study protocol adhered to the principles of the Declaration of Helsinki and received ethical approval from the Dicle University Medical Faculty Ethics Committee for Non-Interventional Studies (Date: 24.12.2025, Approval No: 5). As the study was based on retrospective review of anonymized medical records, the requirement for individual patient informed consent was waived. All procedures followed local and international ethical standards, ensuring patient confidentiality and data protection.

### Clinical, laboratory, and histopathological assessment

Demographic and clinical data—including age, sex, disease duration, and systemic sclerosis (SSc) subtype (limited cutaneous [lcSSc] or diffuse cutaneous [dcSSc])—were retrospectively extracted from electronic medical records. Consecutive patients were evaluated to minimize selection bias. Major organ involvement, particularly interstitial lung disease (ILD), was determined based on prior high-resolution computed tomography (HRCT) reports interpreted by experienced radiologists. Other systemic manifestations were recorded to enable comprehensive comparisons between patients with and without autoimmune hepatitis (AIH).

All patients underwent routine liver function testing as part of a standard clinical follow-up at regular outpatient visits. For the purpose of this study, baseline laboratory values were defined as the earliest available measurements obtained during follow-up prior to any documented diagnosis of AIH. Recorded laboratory parameters included serum alanine aminotransferase (ALT), aspartate aminotransferase (AST), total immunoglobulin G (IgG), and autoantibody profiles (ANA, anti-Scl-70, and anticentromere antibody [ACA]), retrieved from the institutional laboratory database.

Persistent transaminase elevation was defined as ALT and/or AST levels above the upper limit of normal on at least two consecutive measurements over a minimum interval of three months. Patients with persistent elevations and/or suggestive serological findings were referred for hepatology evaluation to confirm or rule out AIH.

To minimize misclassification, cases with liver enzyme elevations temporally associated with hepatotoxic medications—particularly methotrexate—were carefully reviewed. When liver enzyme abnormalities resolved after drug discontinuation or were deemed compatible with drug-induced liver injury (DILI), these patients were excluded from AIH classification.

AIH diagnosis was confirmed by ultrasound-guided percutaneous liver biopsy in all cases. Histopathological features—including interface hepatitis, lymphoplasmacytic infiltration, emperipolesis, and hepatic rosette formation—were documented according to original pathology reports issued by experienced hepatopathologists. Liver fibrosis staging was recorded according to the scoring system applied at the time of diagnosis.

For this study, AIH–SSc overlap was validated only when retrospectively reviewed clinical, serological, and histological findings fulfilled the revised International Autoimmune Hepatitis Group (IAIHG) diagnostic scoring criteria, ensuring accurate differentiation between AIH-positive and AIH-negative groups. This approach strengthens diagnostic rigor and provides a reliable basis for prevalence estimation.

### Statistical analysis

All statistical analyses were performed using IBM SPSS Statistics version 27 (IBM Corp., Armonk, NY, USA). Continuous variables were expressed as mean ± standard deviation (SD) or median with interquartile range (IQR), depending on distribution normality assessed using the Shapiro–Wilk test. Categorical variables were summarized as frequencies and percentages and compared using the Chi-square test or Fisher’s exact test, as appropriate.

Given the low frequency of AIH-SSc overlap (*n* = 8) and the small number of events, conventional logistic regression was considered potentially unreliable due to small-event bias and separation issues. Therefore, Firth’s penalized likelihood logistic regression was applied to estimate adjusted odds ratios (ORs) with 95% confidence intervals (CIs), providing more robust and less biased estimates in the context of rare events. Variables included in the multivariable model were selected based on clinical relevance and univariable analysis results (*p* < 0.10 threshold).

To account for potential confounding by hepatotoxic medications, particularly methotrexate, patients with transaminase elevations temporally associated with drug exposure were carefully reviewed and excluded from AIH classification prior to regression analyses.

To evaluate the diagnostic performance of biochemical markers, receiver operating characteristic (ROC) curve analyses were conducted for alanine aminotransferase (ALT), aspartate aminotransferase (AST), and serum IgG levels. The area under the curve (AUC) was calculated with 95% confidence intervals. Optimal cut-off values were determined using the Youden Index. Sensitivity and specificity were reported with corresponding 95% confidence intervals to provide a fuller picture of diagnostic reliability, as recommended by the reviewers. Missing data were handled using complete-case analysis, as the proportion of missing values was minimal (< 5%). A two-tailed *p*-value < 0.05 was considered statistically significant for all analyses.

## Results

A total of 111 patients with systemic sclerosis (SSc) were included in the study. The mean age of the cohort was 52.4 ± 13.6 years, with a marked female predominance (88.3%). The median disease duration was 7.0 years (IQR 3.0–13.0). Regarding disease subtype, 66.7% of patients had limited cutaneous SSc (lcSSc), while 33.3% had diffuse cutaneous SSc (dcSSc). Autoimmune hepatitis (AIH) was identified in 8 patients, corresponding to an overlap prevalence of 7.2%. The mean age at AIH diagnosis was 46.5 ± 14.2 years. Comparative analyses between patients with and without AIH revealed no statistically significant differences in age (*p* = 0.364), sex (*p* = 0.945), or disease duration (*p* = 0.182). Likewise, the distribution of SSc subtypes and the prevalence of interstitial lung disease (ILD) were similar between the two groups (*p* = 0.795 and *p* = 0.495, respectively). Autoantibody profiles did not differ significantly between groups. Antinuclear antibody (ANA) positivity was nearly universal (100% in AIH-positive vs. 97.1% in AIH-negative patients, *p* = 0.622). Anti–Scl-70 antibodies were detected in 25.0% of patients with AIH compared to 36.9% of those without AIH (*p* = 0.491). Detailed demographic and clinical characteristics are presented in Table 1.

**Table 1:** Comparison of demographic and clinical characteristics of systemic sclerosis patients according to AIH status

Variable	Total cohort (*n* = 111)	AIH (+) (*n* = 8)	AIH (–) (*n* = 103)	*p*-value
Age (years), mean ± SD	52.4 ± 13.6	48.2 ± 12.4	52.7 ± 13.7	0.364
Female sex, *n* (%)	98 (88.3)	7 (87.5)	91 (88.3)	0.945
Disease duration (years), median (IQR)	7.0 (3.0–13.0)	9.5 (4.2–15.0)	6.8 (3.0–12.5)	0.182
Scleroderma subtype, *n* (%)				0.795
• Limited Cutaneous (lcSSc)	74 (66.7)	5 (62.5)	69 (67.0)	
• Diffuse Cutaneous (dcSSc)	37 (33.3)	3 (37.5)	34 (33.0)	
Antibody Profile, *n* (%)				
• ANA Positivity	108 (97.3)	8 (100)	100 (97.1)	0.622
• Anti-Scl-70	40 (36.0)	2 (25.0)	38 (36.9)	0.491
• Anti-centromere	32 (28.8)	3 (37.5)	29 (28.1)	0.574
Organ Involvement, *n* (%)				
• Interstitial Lung Disease (ILD)	68 (61.3)	4 (50.0)	64 (62.1)	0.495
• Raynaud's Phenomenon	106 (95.5)	8 (100)	98 (95.1)	0.999
• Pulmonary Hypertension	22 (19.8)	2 (25.0)	20 (19.4)	0.655
• Gastrointestinal Involvement	41 (36.9)	3 (37.5)	38 (36.9)	0.972

Continuous variables are presented as mean ± standard deviation or median (interquartile range, IQR), as appropriate. Categorical variables are expressed as number (percentage). Comparisons between groups were performed using the Student’s *t*-test or Mann–Whitney *U* test for continuous variables and the chi-square test or Fisher’s exact test for categorical variables, as appropriate. A *p* value < 0.05 was considered statistically significant

*AIH *autoimmune hepatitis, *ANA *antinuclear antibody, *lcSSc *limited cutaneous systemic sclerosis, *dcSSc *diffuse cutaneous systemic sclerosis, *ILD *interstitial lung disease

Among patients with AIH overlap, laboratory evaluation demonstrated elevated liver transaminase levels, with a median AST of 58.0 U/L (IQR 42.0–112.5) and ALT of 64.5 U/L (IQR 38.0–124.0). Hypergammaglobulinemia was a prominent finding; the mean serum IgG level was 1845 ± 412 mg/dL, and 75.0% of patients had IgG levels exceeding 1600 mg/dL. Liver biopsy was performed in all eight patients with suspected AIH. Histopathological examination revealed interface hepatitis in all cases (100%). Additional characteristic findings included plasma cell infiltration in 75.0% and hepatocyte rosette formation in 37.5% of biopsies. Cirrhotic changes were identified in one patient (12.5%) at the time of AIH diagnosis.

To address the potential risk of small-sample bias and separation due to the low frequency of AIH cases (*n* = 8), Firth’s penalized likelihood logistic regression was employed to identify factors associated with AIH overlap (Table 2). In univariate analyses, no significant associations were observed between AIH overlap and demographic or clinical variables, including age (OR 0.98, 95% CI 0.92–1.04; *p* = 0.452), female sex (OR 0.85, 95% CI 0.12–6.42; *p* = 0.910), or the presence of interstitial lung disease (OR 0.65, 95% CI 0.18–2.30; *p* = 0.510). Similarly, in the multivariate model, neither the diffuse cutaneous SSc subtype (aOR 1.28, 95% CI 0.30–5.95; *p* = 0.720) nor anti–Scl-70 antibody positivity (aOR 0.48, 95% CI 0.09–2.50; *p* = 0.380) emerged as independent predictors of AIH overlap. Overall, these findings suggest that the occurrence of AIH overlap in patients with SSc did not demonstrate statistically significant associations with the evaluated demographic characteristics, clinical phenotypes, and autoantibody profiles.

**Table 2:** Factors associated with autoimmune hepatitis overlap in patients with systemic sclerosis: Firth’s penalized logistic regression analysis

Variable	Univariate OR (95% CI)	*p*-value	Multivariate aOR (95% CI)	*p*-value
Age (years)	0.98 (0.92–1.04)	0.452	—	—
Female sex	0.85 (0.12–6.42)	0.910	—	—
Diffuse cutaneous SSc	1.15 (0.25–4.80)	0.812	1.28 (0.30–5.95)	0.720
Interstitial lung disease	0.65 (0.18–2.30)	0.510	0.60 (0.15–2.60)	0.540
Anti–Scl-70 positivity	0.55 (0.10–2.80)	0.480	0.48 (0.09–2.50)	0.380
Immunosuppressive therapy	1.65 (0.20–14.20)	0.620	—	—

Odds ratios (ORs) and adjusted odds ratios (aORs) are presented with 95% confidence intervals (CIs). Variables with clinical relevance or a *p* value < 0.20 in univariate analysis were included in the multivariate model. A *p* value < 0.05 was considered statistically significant

*AIH *autoimmune hepatitis, *SSc *systemic sclerosis, *OR *odds ratio, *aOR *adjusted odds ratio, *CI *confidence interval

**Fig. 1:**
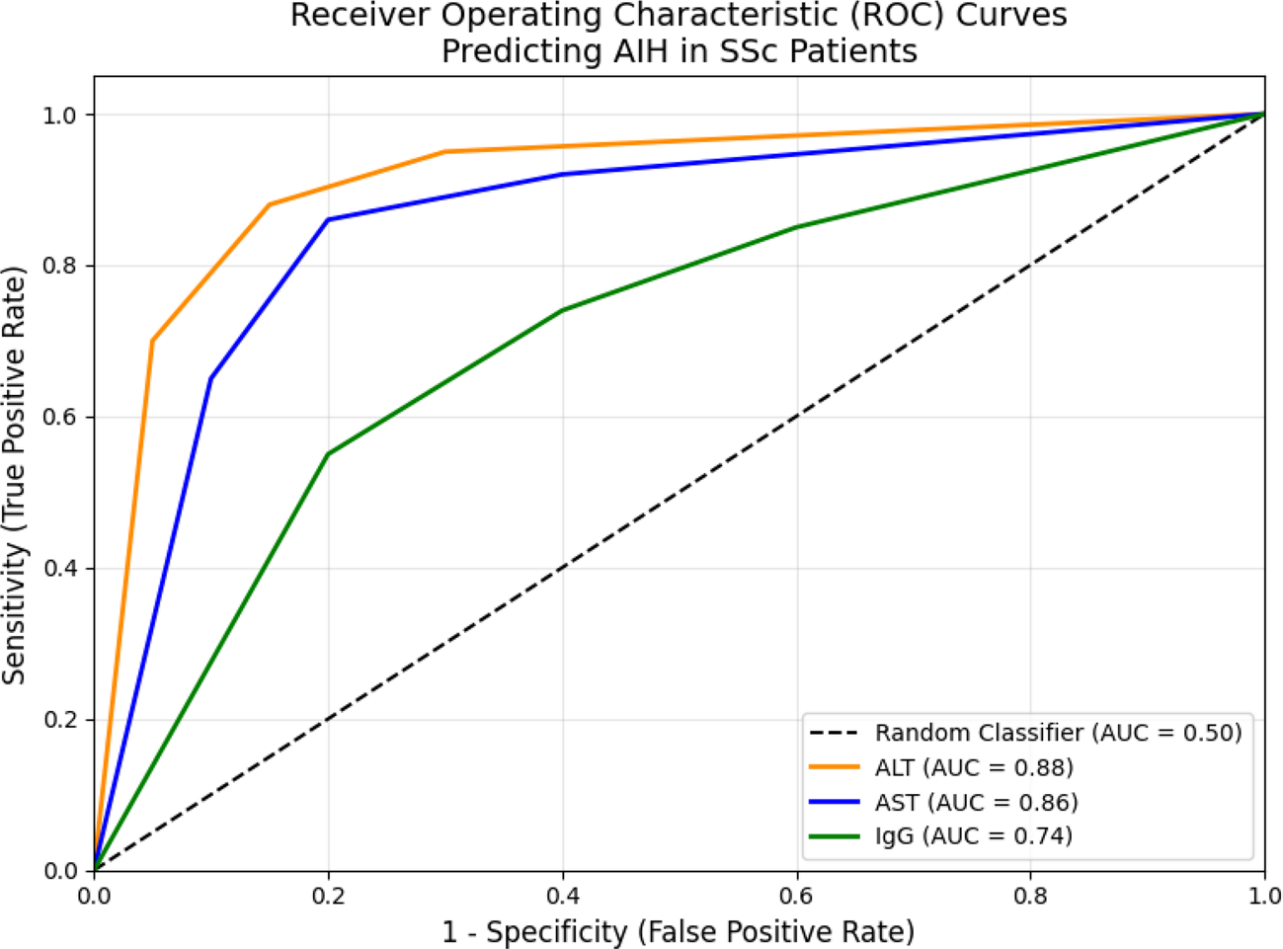
Receiver Operating Characteristic (ROC) curve analysis of laboratory parameters for predicting Autoimmune Hepatitis (AIH) overlap in Systemic Sclerosis patients. The figure illustrates the diagnostic performance of ALT (AUC: 0.88, orange), AST (AUC: 0.86, blue), and IgG levels (AUC: 0.74, green). The dashed diagonal line represents the performance of a random classifier. ALT demonstrated the highest discriminatory power for AIH screening in this cohort

To evaluate the screening performance of laboratory parameters for AIH overlap, receiver operating characteristic (ROC) curve analysis was performed (Fig. 1). Alanine aminotransferase (ALT) demonstrated the highest diagnostic accuracy, with an area under the curve (AUC) of 0.88 (95% CI 0.76–0.99; *p* < 0.001). At an optimal cut-off value of > 34.5 U/L, ALT yielded a sensitivity of 87.5% (95% CI: 47.3–99.7%) and a specificity of 85.4% (95% CI: 76.0–92.0%). Aspartate aminotransferase (AST) also showed strong discriminatory ability (AUC 0.86, 95% CI 0.72–0.99; *p* < 0.001), whereas serum IgG levels exhibited moderate diagnostic performance (AUC 0.74, 95% CI 0.58–0.89; *p* = 0.015). Detailed diagnostic indices, including optimal cut-off values, sensitivities, and specificities, are summarized in Table 3.

**Table 3:** Diagnostic performance of laboratory markers for autoimmune hepatitis prediction

Parameter	AUC (95% CI)	Optimal Cut-off	Sensitivity (95% CI)	Specificity (95% CI)	*p*-value
ALT (U/L)	0.88 (0.76–0.99)	> 34.5	87.5% (95% CI: 47.4–99.7%)	85.4% (95% CI: 76.7–91.8%)	< 0.001
AST (U/L)	0.86 (0.72–0.99)	> 38.0	75.0% (95% CI: 34.9–96.8%)	84.5% (95% CI: 75.5–91.0%)	< 0.001
IgG (mg/dL)	0.74 (0.58–0.89)	> 1640	62.5% (95% CI: 24.5–91.5%)	81.2% (95% CI: 71.6–88.7%)	0.015

The diagnostic performance of each laboratory marker was assessed using Receiver Operating Characteristic (ROC) curve analysis. Optimal cut-off values were determined based on the Youden Index (J = Sensitivity + Specificity − 1), maximizing the combined sensitivity and specificity for each parameter. Among the markers evaluated, alanine aminotransferase (ALT) demonstrated the highest discriminatory power, with an area under the curve (AUC) of 0.88 (95% CI: 0.76–0.99), reflecting excellent diagnostic accuracy according to conventional classification criteria. At the optimal cut-off of > 34.5 U/L, ALT achieved a sensitivity of 87.5% (95% CI: 47.4–99.7%) and a specificity of 85.4% (95% CI: 76.7–91.8%), providing a practical and reliable non-invasive screening tool. Aspartate aminotransferase (AST) also showed strong discriminatory ability, with an AUC of 0.86 (95% CI: 0.72–0.99), and sensitivity and specificity of 75.0% (95% CI: 34.9–96.8%) and 84.5% (95% CI: 75.5–91.0%), respectively. Immunoglobulin G (IgG) demonstrated moderate diagnostic performance, with an AUC of 0.74 (95% CI: 0.58–0.89) and sensitivity and specificity of 62.5% (95% CI: 24.5–91.5%) and 81.2% (95% CI: 71.6–88.7%). The associated *p*-values indicate that the predictive performance of all markers significantly exceeded chance (*p* < 0.05). Overall, ALT and AST exhibited superior precision and clinical utility compared with IgG, supporting their role as first-line biochemical indicators for identifying AIH overlap in SSc patients. All laboratory measurements were obtained from baseline evaluations conducted at the time of clinical suspicion or routine screening

*AUC *area under the curve, *CI *confidence interval, *ALT *alanine aminotransferase, *AST *aspartate aminotransferase, *IgG *immunoglobulin G

Methotrexate exposure was documented in 21 of 111 patients (18.9%). Among patients with ALT levels > 34.5 U/L (*n* = 21), six were receiving methotrexate therapy. Importantly, none of the AIH-positive patients had prior methotrexate exposure. The positive predictive value of ALT > 34.5 U/L for AIH diagnosis was 33.3% (7/21), indicating that approximately one-third of SSc patients exceeding this threshold were ultimately diagnosed with biopsy-confirmed AIH. These findings highlight that while methotrexate-associated liver enzyme elevations must be considered, persistent ALT elevation above the identified threshold warrants evaluation for AIH, particularly when drug-related temporal association is absent.

## Discussion

The coexistence of systemic sclerosis (SSc) and autoimmune hepatitis (AIH) represents a rare but clinically meaningful overlap that remains far less explored than the well-established association between SSc and primary biliary cholangitis (PBC) [14]. In this study, we provide a comprehensive evaluation of AIH in a well-characterized SSc cohort, identifying a prevalence of 7.2%. This rate exceeds the 1.5% reported by Assassi et al. and the 1–2% generally cited in large scleroderma registries, yet is consistent with recent European data suggesting that hepatic autoimmune involvement may be underrecognized when systematic screening is not employed [15]. Geographic and genetic heterogeneity, differences in diagnostic rigor, and historical diagnostic overshadowing by cholestatic liver disease likely contribute to the variability in reported prevalence [15, 16].

A key finding of our study is the apparent phenotypic independence of autoimmune hepatitis (AIH) within the systemic sclerosis (SSc) disease spectrum. Contrary to the common assumption that visceral involvement clusters with markers of severe systemic disease—such as diffuse cutaneous SSc (dcSSc), anti-Scl70 positivity, or advanced pulmonary involvement—we found no significant association between AIH and skin subtype, disease duration, or major organ complications including interstitial lung disease (ILD) [17]. These results suggest that hepatic involvement in SSc does not represent a downstream consequence of systemic fibrosis, but rather reflects a parallel, organ-specific autoimmune process. This observation carries important clinical implications, as it underscores that liver surveillance should not be restricted to patients with aggressive or advanced SSc phenotypes. Even individuals with limited cutaneous SSc (lcSSc) and otherwise indolent disease courses may be at risk, supporting the need for universal biochemical monitoring [17].

From a serological standpoint, the predictive value of SSc-specific autoantibodies for hepatic involvement remains uncertain [18]. Although previous studies have suggested potential links between anti-centromere antibodies (ACA) and autoimmune liver diseases—predominantly primary biliary cholangitis (PBC)—our data did not demonstrate a significant association between any SSc-related autoantibody profile and autoimmune hepatitis (AIH) [18]. This finding is in line with prior observations by Marie et al., indicating that while SSc-specific antibodies are valuable for stratifying cutaneous and pulmonary risk, they offer limited utility in anticipating hepatic overlap [3]. To robustly address the statistical challenges imposed by the rarity of AIH cases (*n* = 8), we employed Firth’s penalized likelihood regression, an approach specifically designed to mitigate bias in rare-event analyses. Using this method, we confirmed that none of the conventional clinical or serological markers of SSc independently predicted AIH, reinforcing the need for direct, liver-focused screening strategies.

The most clinically actionable insight from our study lies in the diagnostic performance of non-invasive biochemical markers. Although the International Autoimmune Hepatitis Group (IAIHG) scoring system remains the diagnostic gold standard, its complexity limits its practicality in routine rheumatology practice [19]. Our ROC analysis demonstrated that alanine aminotransferase (ALT) is a highly effective screening tool in this context, with an AUC of 0.88, significantly outperforming both AST and IgG. While elevated IgG is a hallmark of autoimmune hepatitis (AIH), its specificity may be compromised in systemic sclerosis (SSc) due to background polyclonal hypergammaglobulinemia driven by chronic immune activation [20]. In contrast, an ALT threshold of > 34.5 U/L achieved a favorable balance between sensitivity (87.5%) and specificity (85.4%), offering a simple and cost-effective “red flag” to prompt further hepatologic evaluation, including liver-specific autoantibody testing and biopsy when indicated.

Importantly, 21 of 111 patients in our cohort (18.9%) had prior methotrexate (MTX) exposure. Among patients with ALT levels > 34.5 U/L, six were receiving MTX therapy, while none of the AIH-positive patients had prior MTX exposure, suggesting that elevated ALT in our cohort is unlikely to reflect MTX-induced liver injury.

Early recognition of autoimmune hepatitis (AIH) is particularly critical given its potential for rapid progression if left untreated [21]. Unlike primary biliary cholangitis (PBC), which often follows a slowly progressive course, AIH can evolve into aggressive lobular hepatitis and cirrhosis in the absence of timely immunosuppressive therapy [21]. Our findings support a proactive surveillance model in which persistent transaminase elevations—rather than overt clinical signs of advanced liver disease—trigger diagnostic escalation. Integrating routine ALT monitoring into standard systemic sclerosis (SSc) follow-up protocols may therefore alter the long-term hepatic prognosis of affected patients [21, 22].

A major strength of this study is the stringent diagnostic approach employed for autoimmune hepatitis, with all AIH cases confirmed by liver biopsy in accordance with International Autoimmune Hepatitis Group (IAIHG) criteria. This histopathological confirmation minimizes diagnostic misclassification, a common limitation in registry-based or serology-driven studies of hepatic involvement in systemic sclerosis. Furthermore, the use of Firth’s penalized likelihood regression allowed for robust analysis despite the rarity of AIH-SSc overlap, ensuring more reliable estimation of associations in a small-event setting. Importantly, our findings translate directly into clinical practice by proposing a simple, non-invasive biochemical screening threshold that can be readily implemented in routine rheumatology follow-up.

Several limitations warrant consideration. The retrospective, single-center design may introduce selection bias and limit generalizability to broader, ethnically diverse populations. Additionally, the small number of AIH cases, while expected given the rarity of this overlap, reduces statistical power and necessitates cautious interpretation of predictive estimates. Finally, although our analysis primarily focused on diagnostic performance, the limited sample size and single-center nature constrain conclusions regarding risk factors, phenotypic associations, and treatment response. Future multicenter, prospective studies with larger cohorts are needed to validate our findings, refine screening thresholds, and determine whether SSc-related features influence long-term hepatic outcomes in patients with AIH overlap.

Future studies should aim to validate these findings in large, multicenter, prospective cohorts to confirm the diagnostic performance of ALT and refine optimal screening thresholds across diverse populations. Longitudinal investigations are also needed to determine whether systemic sclerosis–specific features influence treatment response, disease progression, or long-term hepatic outcomes in AIH overlap patients. Additionally, exploration of shared genetic susceptibility—particularly HLA-DR3 and DR4 alleles known to be associated with AIH—may provide mechanistic insight into why a subset of SSc patients develops this overlap. Such efforts could ultimately inform risk-stratified screening strategies and further optimize early detection and management of hepatic involvement in systemic sclerosis.

## Conclusion

In conclusion, our study demonstrates that autoimmune hepatitis is a rare but clinically distinct overlap syndrome in systemic sclerosis that appears to manifest independently of established disease phenotypes, cutaneous subtypes, or autoantibody profiles. This independent nature suggests that AIH does not merely follow the severity of systemic fibrotic involvement but arises from distinct autoimmune pathways, necessitating a high index of suspicion across the entire SSc spectrum. Our findings highlight the critical role of routine biochemical monitoring, with ALT emerging as a highly effective non-invasive screening tool to guide clinical decision-making. Importantly, in our cohort, prior methotrexate (MTX) exposure did not confound the diagnostic performance of ALT, supporting its specificity for AIH screening in SSc patients.

Furthermore, based on our findings, we propose a practical monitoring approach: regular ALT assessment for all SSc patients, with elevations above 34.5 U/L prompting consideration of AIH evaluation, including liver-specific autoantibody testing and biopsy when indicated. While the established diagnostic thresholds in this study provide a practical framework for early detection, further large-scale, prospective, and multi-center studies are required to validate these risk factors, refine ALT cut-offs, and optimize structured liver monitoring protocols in broader SSc populations.

## Data Availability

The datasets generated and analyzed during this study are available from the corresponding author upon reasonable request.
